# Associations of the neutrophil to lymphocyte ratio with intracranial artery stenosis and ischemic stroke

**DOI:** 10.1186/s12883-021-02073-3

**Published:** 2021-02-05

**Authors:** Liang-Yu Huang, Fu-Rong Sun, Jian-Jun Yin, Ya-Hui Ma, Hong-Qi Li, Xiao-Ling Zhong, Jin-Tai Yu, Jing-Hui Song, Lan Tan

**Affiliations:** 1 Department of Neurology, Qingdao Municipal Hospital, Qingdao University, Qingdao, 266071 China; 2 Department of Neurology, Qingdao Hiser Hospital, Qingdao University, Qingdao, China; 3 Department of Neurology and Institute of Neurology, Huashan Hospital, Shanghai Medical College, Fudan University, 12th Wulumuqi Zhong Road, Shanghai, 200040 China; 4 Department of Neurology, Qingdao Central Hospital, Qingdao University, Qingdao, China; 5 Department of Neurology, The Affiliated Hospital of Qingdao University, Qingdao, China

**Keywords:** Intracranial artery stenosis, Ischemic stroke, Neutrophil to lymphocyte ratio, Mediation analysis

## Abstract

**Background:**

The neutrophil-to-lymphocyte ratio (NLR) has emerged as an inflammatory marker. However, the associations of NLR with intracranial artery stenosis (ICAS) and ischemic stroke remain unclear. This study aimed to examine the associations of NLR with ICAS and ischemic stroke among a large and high-risk population.

**Methods:**

Participants with records of clinical characteristics were prospectively recruited from the Neurology Department and Health & Physical Examination Center of Qingdao Municipal Hospital. Logistic regression analysis was used to examine the associations of NLR with ICAS and ischemic stroke. Moreover, we also conducted parametric mediation analysis to estimate the effect of NLR on the risk of ischemic stroke mediated through ICAS.

**Results:**

A total of 2989 participants were enrolled in this study. After adjusting for covariates, NLR (OR = 1.125, 95%CI 1.070–1.183) and ICAS (OR = 1.638, 95%CI 1.364–1.967) were significantly associated with ischemic stroke. Compared with the first quartile NLR, the second, third and fourth quartiles NLR were independent risk predictors for ischemic stroke (*P* for trend < 0.001); the third and fourth quartiles were independent predictors for ICAS (*P* for trend < 0.001). The mediation analysis showed that ICAS partially mediated the association between NLR and ischemic stroke, accounting for 14.4% of the total effect (*P* < 0.001).

**Conclusions:**

NLR was significantly associated with ICAS and ischemic stroke. Besides, ICAS partially mediated the association between NLR and ischemic stroke.

## Background

Inflammation has been found to play an vital role in atherosclerosis [1], with various inflammatory parameters being associated with artery stenosis [2, 3] as well as ischemic events [4–6]. Neutrophil to lymphocyte ratio (NLR) is an indicator of overall systemic inflammatory status in the Asian population and NLR can be of prognostic value in several disorders [7].

Similar to other inflammatory factors, NLR not only serves as an independent risk factor of atherosclerosis in carotid arteries, coronary arteries, and even peripheral arteries [8–11], but also helps to predict the occurrence and prognosis of stroke [12–15] . On the spectrum of atherosclerosis, intracranial artery stenosis (ICAS) was also associated with the inflammatory process. However, the association of NLR with ICAS remains unclear. Besides, research focusing on the risk of ischemic stroke with the presence of high NLR and ICAS is limited, while the ICAS is one of the most common causes of ischemic stroke [16, 17]. Moreover, the pathogenesis of cerebral atherosclerosis urgently needs to be well addressed for the high prevalence and pathogenicity of ICAS [18, 19]. Since the NLR is adjustable by controlling inflammation, determining the impact of NLR on the risks of ICAS and ischemic stroke is desired. Such work may contribute to better understanding on the pathophysiological mechanism of ICAS and ischemic stroke as well as exploring new therapeutic targets. Accordingly, this study aimed to examine the differential associations of NLR with ICAS and ischemic stroke among a large and high-risk population.

## Methods

### Study design and participants

Study subjects were prospectively recruited from the Neurology Department and Health Screening Center of Qingdao Municipal Hospital from January 2014 to June 2018. Patients in the department of neurology were diagnosed as suspected acute ischemic stroke. Ischemic stroke is defined as transient ischemic attack (TIA) or acute ischemic stroke (within 7 days of onset). The non-stroke controls enrolled from health screening center were free from serious health problems as well as TIA and acute ischemic stroke. Exclusion criteria for all participants comprised (1) less than 40 years old; (2) incomplete vascular imaging and laboratory tests; (3) evidences of cardioembolic propensity such as history of atrial fibrillation, valvular heart disease, and underwent replacement; (4) intracranial and external artery dissection, arteritis, moyamoya disease, muscular fiber dysplasia; (5) infection, nausea, tumor, chronic liver disease, and renal insufficiency; (6) intracranial or extracranial cerebral artery stenting or balloon angioplasty; (7) hemorrhagic stroke. Written informed consent form was obtained from all participants or their guardians. This study was conducted in accordance with the Declaration of Helsinki and approved by the Institutional Ethics Committees of Qingdao Municipal Hospital.

Finally, a total of 2989 participants who underwent magnetic resonance angiography (MRA) were enrolled in this study, including 1867 participants with ischemic stroke as well as 1122 participants without stroke.

### Laboratory measurements

Fasting blood samples for biochemical analysis were collected within 24 h after the hospital admission and were measured at the central laboratory of the Qingdao Municipal Hospital by using an automated analytical platform (Beckman Coulter AU5800: Beckman Coulter Inc. Brea, CA, USA). Fasting blood-glucose and blood fats (high-density lipoprotein, low-density lipoprotein, total cholesterol and triglyceride) were measured using blood samples drawn at approximately 7 a.m. after an overnight fast. Neutrophil count and lymphocyte count in EDTA-anticoagulated whole-blood samples from venipuncture were determined with automated particle counters within the first 24 h after admission. NLR was calculated as the ratio of neutrophil count to lymphocyte count (NLR = Neutrophil count/Lymphocyte count).

### Imaging assessment

All the included participants had completed magnetic resonance imaging (MRI) and 3D-time-offlight MRA by 3.0-T magnetic resonance to evaluate intracranial artery status. Besides, ultrasonography examination contrast-enhanced MRA, or computed tomography angiography (CTA) were performed for evaluating extracranial carotid arteries. According to Warfarin-Aspirin Symptomatic Intracranial Disease (WASID) trial criteria [20], the presence of ICAS was defined as the presence of 50–99% stenosis or the occlusion of intracranial arteries including intracranial section of internal carotid artery (ICA), M1/M2 segment of middle cerebral artery (MCA), A1/A2 segment of anterior cerebral artery (ACA), P1/P2 segment of posterior cerebral artery (PCA), V5 segment of vertebral artery (VA), and basilar artery (BA). Two trained investigators who were blinded to clinical information independently evaluated the presence of ICAS, and a third neuroradiologist was consulted in discussion when disagreements emerging.

### Assessment of clinical risk factors

The clinical characteristics including age, gender, medical histories of coronary heart disease (CHD), hypertension, diabetes mellitus, stroke and drinking were collected in face-to-face interviews by physicians after referral or hospital admission and cross referenced with primary care records. Before the measurement of blood pressure, subjects were required to rest for at least 5 min, and no less than twice measurements were conducted to obtain stable blood pressures. Hypertension was defined as having an average systolic pressure ≥ 140 mmHg or an average diastolic pressure ≥ 90 mmHg on≥3 occasions or taking antihypertensive drugs. Drinking was considered as previous history of or current moderate to severe alcohol consumption (> 168 g/week). Smoking was defined as a patient who had smoked continuously for 6 months ≥1 cigarette a day. Diabetes mellitus was defined as having a fasting serum glucose level ≥ 7 mg/dL, a non-fasting serum glucose level ≥ 11.1 mg/dL, or use of hypoglycemic agent. CHD was defined as newly diagnosed or a known history of coronary artery disease.

### Statistical analysis

The main analyses were conducted in 2 steps. Firstly, multivariable logistic regression was used to estimate the odds ratio (OR) of NLR for ischemic stroke. Unadjusted ORs were calculated from a univariable model (model 1). In model 2, age, gender, systolic blood pressure, coronary heart disease, smoking, drinking, glucose, and total cholesterol-adjusted OR was estimated. In model 3, the presence of ICAS was added to model 2. Secondly, to estimate the mediated proportion of the effect of NLR on ischemic stroke, a parametric mediation analysis was conducted. In this mediation analysis, the effect was defined in terms of the difference in regression coefficient by logistic regression models. The total effect (TE) of NLR on ischemic stroke was estimated and was divided into direct effect and the mediated effect (ME) through ICAS. Additionally, the mediated proportion (ie, ME/TE) was estimated. In the mediation analysis, age, gender, systolic blood pressure, coronary heart disease, smoking, drinking, glucose and total cholesterol were adjusted in the logistic regression model.

As for characteristics of the study participants, the distributions of continuous variables were tested by Kolmogorov-Smirnov tests. Continuous variables without normal distribution were expressed as median (50th) values and interquartile ranges (25th and 75th), compared by the Mann–Whitney U-test. Categorical variables were presented as frequency and percentage, analyzed by χ2 test. For the tabulation and logistic regression analysis, SPSS software version 23.0 for Windows (SPSS Inc., Chicago, IL) was used. Mediation analysis was performed using R 3.62version (“lm” function in the package “mediation”). A 2-tailed *P* value < 0.05 was considered statistically significant.

## Results

### Baseline characteristics of study subjects

A total of 2989 individuals (mean age 68.03 ± 11.37 years and 39.00% female) were included in this analysis, among whom 1867 patients with acute ischemic stroke (612 with ICAS and 1255 without ICAS) and 1122 non-stroke controls (239 with ICAS and 883 without ICAS). Clinical characteristics are presented in Table 1. The median NLR level of all participants was 1.92 (interquartile range 1.43–2.76). NLR levels (median 2.01, interquartile range 1.49–3.00) were higher in patients with stroke than those (median 1.76, interquartile range 1.33–2.44, *p* < 0.001) in controls without stroke. Furthermore, Levels of systolic blood pressure (SBP), diastolic blood pressure (DBP), glucose, low-density lipoprotein (LDL), levels of total cholesterol (TC), and triglyceride (TG); the prevalence of coronary heart disease (CHD) and ICAS; and drinking and smoking frequency were higher in the stroke group than in the non-stroke group.

**Table 1:** Baseline characteristics and neutrophil-to-lymphocyte ratio according to status of ischemic stroke

	Total participants included (*n* = 2989)	All participants with stroke (*n* = 1867)	All participants without stroke (*n* = 1122)	*P*
Age, mean ± SD, years	68.03 (11.37)	67.96 (11.57)	68.16 (11.04)	0.717
Female	1165 (38.98)	658 (35.26)	507 (45.23)	0.000^*^
SBP, median [IQR], mmHg	146 [130–160]	150 [135–165]	140 [130–155]	0.000^*^
DBP, median [IQR], mmHg	82 [80–90]	85 [80–92]	80 [78–90]	0.000^*^
Hematological examinations				
Glucose, median (IQR), mmol/L	5.32 (4.67–6.91)	5.47 (4.70–7.43)	5.16 (4.62–6.36)	0.000^*^
HDL, median (IQR), mmol/L	1.12 (0.95–1.33)	1.12 (0.95–1.31)	1.12 (0.95–1.35)	0.200
LDL, median (IQR), mmol/L	2.98 (2.40–3.57)	3.04 (2.48–3.66)	2.85 (2.29–3.43)	0.000^*^
TC, median (IQR), mmol/L	4.88 (4.10–5.70)	4.95 (4.17–5.83)	4.71 (3.97–5.33)	0.000^*^
TG, median (IQR), mmol/L	1.29 (0.95–1.76)	1.30 (0.97–1.78)	1.27 (0.91–1.73)	**0.035**^**†**^
Neutrophils, median (IQR), (10^9^/L)	3.83 (2.99–4.96)	4.05 (3.17–5.36)	3.49 (2.78–4.37)	0.000^*^
Lymphocytes, median (IQR), (10^9^/L)	1.94 (1.52–2.41)	1.94 (1.52–2.43)	1.94 (1.51–2.40)	0.467
NLR, median (IQR)	1.92 (1.43–2.76)	2.01 (1.49–3.00)	1.76 (1.33–2.44)	0.000^*^
Medical history				
Hypertension, n (%)	2370 (79.29)	1476 (79.06)	894 (79.68)	0.685
Diabetes mellitus, n (%)	1079 (36.10)	694 (37.17)	385 (34.31)	0.115
Stroke, n (%)	711 (23.79)	449 (24.05)	262 (23.35)	0.000^*^
CHD, n (%)	1141 (38.17)	628 (33.64)	513 (45.72)	0.000^*^
Smoking, n (%)	1015 (33.96)	690 (36.96)	325 (28.97)	0.000^*^
Drinking, n (%)	716 (23.95)	492 (26.35)	224 (19.96)	0.000^*^
ICAS, n (%)	851 (28.47)	612 (32.78)	239 (21.30)	0.000^*^

*Abbreviations*: *SBP* systolic blood pressure, *DBP* diastolic blood pressure, *HDL* high-density lipoprotein, *LDL* low-density lipoprotein, *TC* total cholesterol, *TG* triglyceride, *NLR* neutrophil to lymphocyte ratio, *CHD* coronary heart disease, *SD* standard deviation, *IQR* interquartile range, P* for comparisons between stroke and non-stroke groups

^*^P < 0.001; ^†^*P* < 0.05

### Associations of NLR with ICAS and ischemic stroke

**Fig. 1:**
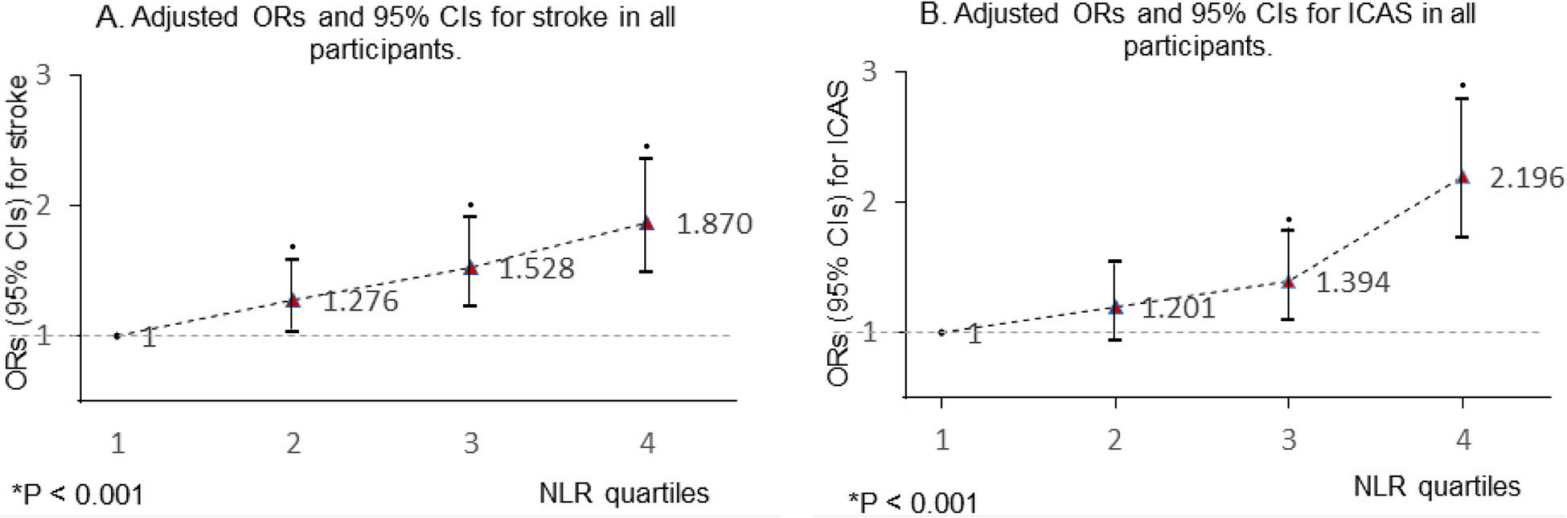
Multinomial-adjusted ORs and 95% CIs for ICAS and ischemic stroke according to NLR quartiles. NLR was significantly associated with ischemic stroke in the second, third and fourth quartiles. NLR was significantly associated with ICAS in the third and fourth quartiles in all participants. (the first NLR quartile as reference). •*p* < 0.05; *p: *p* value for trend

The results of the relationship between NLR and Ischemic stroke by logistic regression analysis are shown in Table 2. The univariate OR was 1.163 (95%CI 1.107–1.233) in model 1. After adjustment for age, gender, systolic blood pressure (SBP), coronary heart disease (CHD), smoking, drinking, glucose, total cholesterol (TC), the ischemic stroke OR with higher NLR was 1.144 (95%CI 1.088–1.203). In model 3, including all covariates (adjusted in model 2) and ICAS (ie, a mediator), NLR (OR = 1.125, 95%CI 1.070–1.183) and ICAS (OR = 1.638, 95%CI 1.364–1.967) were significantly associated with ischemic stroke. Despite the adjustment for ICAS, NLR remained independently associated with ischemic stroke. As shown in Table 3, NLR was also an independent risk factor for ICAS after adjusting age; gender; SBP; history of hypertension, diabetes mellitus, CHD, smoking and drinking; glucose; and high-density lipoprotein. The covariates and ICAS-adjusted results for ICAS and ischemic stroke according to NLR quartiles are shown in Fig. 1. Compared with the first quartile, the second (OR = 1.276, 95%CI 1.030–1.580), third (OR = 1.528, 95%CI 1.228–1.903) and fourth (OR = 1.870, 95%CI 1.487–2.353) quartiles were independent risk predictors for ischemic stroke (*P* for trend < 0.001); the third (OR = 1.394, 95%CI 1.092–1.779) and forth (OR = 2.196, 95%CI 1.729–2.790) quartiles were independent predictors for ICAS (*P* for trend < 0.001).

**Table 2:** Logistic regression estimation of the effect on ischemic stroke with neutrophil to lymphocyte ratio at baseline

	Model 1 OR (95% CI)	*P*	Model 2 OR (95% CI)	*P*	Model 3	*P*
					OR (95% CI)	
Age	0.999 (0.992–1.005)	0.640	1.005 (0.998–1.013)	0.145	1.004 (0.996–1.011)	0.304
Gender	0.660 (0.568–0.768)	0.000^*^	0.723 (0.595–0.878)	**0.001**^**†**^	0.706 (0.580–0.858)	0.000^*^
Systolic blood pressure	1.018 (1.014–1.021)	0.000^*^	1.016 (1.013–1.020)	0.000^*^	1.016 (1.012–1.020)	0.000^*^
Hypertension	0.963 (0.802–1.156)	0.685				
Diabetes mellitus	1.133 (0.970–1.322)	0.115				
Stroke history	1.039 (0.873–1.237)	0.664				
Coronary heart disease	0.602 (0.517–0.700)	0.000^*^	0.578 (0.490–0.681)	0.000^*^	0.575 (0.488–0.679)	0.000^*^
Smoking	1.438 (1.226–1.686)	0.000^*^	1.216 (0.975–1.517)	0.083	1.226 (0.982–1.530)	0.072
Drinking	1.434 (1.199–1.716)	0.000^*^	1.055 (0.838–1.327)	0.649	1.028 (0.816–1.295)	0.814
Glucose	1.090 (1.057–1.124)	0.000^*^	1.081 (1.047–1.116)	0.000^*^	1.072 (1.038–1.107)	0.000^*^
High-density lipoprotein	0.938 (0.780–1.127)	0.494				
Low-density lipoprotein	1.001 (0.981–1.021)	0.922				
Triglyceride	1.056 (0.984–1.134)	0.132				
Total cholesterol	1.173 (1.104–1.245)	0.000^*^	1.161 (1.088–1.240)	0.000^*^	1.170 (1.095–1.250)	0.000^*^
Neutrophil to lymphocyte ratio	1.163 (1.107–1.233)	0.000^*^	1.144 (1.088–1.203)	0.000^*^	1.125 (1.070–1.183)	0.000^*^
Intracranial artery stenosis					1.638 (1.364–1.967)	0.000^*^

Model 1: crude; Model 2: adjusted for age, gender, systolic blood pressure, coronary heart disease, smoking, drinking, glucose, total cholesterol

Model 3: model 2 + adjusted for intracranial artery stenosis

^*^P < .001

^†^*P* < .05

**Table 3:** Logistic regression estimation of the effect on intracranial artery stenosis with neutrophil to lymphocyte ratio at baseline

	Model 1 OR (95% CI)	*P*	Model 2 OR (95% CI)	*P*
Age	1.022 (1.015–1.029)	0.000^*^	1.020 (1.012–1.028)	0.000^*^
Female	1.190 (1.012–1.398)	**0.036**^**†**^	1.290 (1.050–1.585)	**0.015**^**†**^
Systolic blood pressure	1.008 (1.005–1.012)	0.000^*^	1.005 (1.002–1.009)	**0.005**^**†**^
Hypertension	1.387 (1.129–1.704)	**0.002**^**†**^	1.192 (0.952–1.493)	0.125
Diabetes mellitus	1.605 (1.364–1.888)	0.000^*^	1.292 (1.052–1.587)	**0.015**^**†**^
Coronary heart disease	1.190 (1.011–1.399)	**0.036**^**†**^	0.943 (0.790–1.126)	0.516
Smoking	0.837 (0.706–0.992)	**0.040**^**†**^	0.920 (0.728–1.163)	0.487
Drinking	1.001 (0.831–1.206)	0.989	1.354 (1.063–1.723)	**0.014**^**†**^
Glucose	1.086 (1.056–1.117)	0.000^*^	1.053 (1.017–1.091)	**0.003**^**†**^
High-density lipoprotein	0.689 (0.539–0.880)	**0.003**^**†**^	0.646 (0.495–0.841)	**0.001**^**†**^
Low-density lipoprotein	0.984 (0.927–1.044)	0.584	–	–
Triglyceride	0.953 (0.882–1.029)			
Total cholesterol	0.969 (0.910–1.031)	0.319	–	–
Neutrophil to lymphocyte ratio	1.183 (1.133–1.234)	0.000^*^	1.158 (1.108–1.210)	0.000^*^

Model 1: Crude; Model 2: Adjusted for age, gender, systolic blood pressure, hypertension, diabetes mellitus, coronary heart disease, smoking, drinking, glucose, high-density lipoprotein

^*^P < .001

^†^*P* < .05

### Mediation analysis

**Fig. 2:**
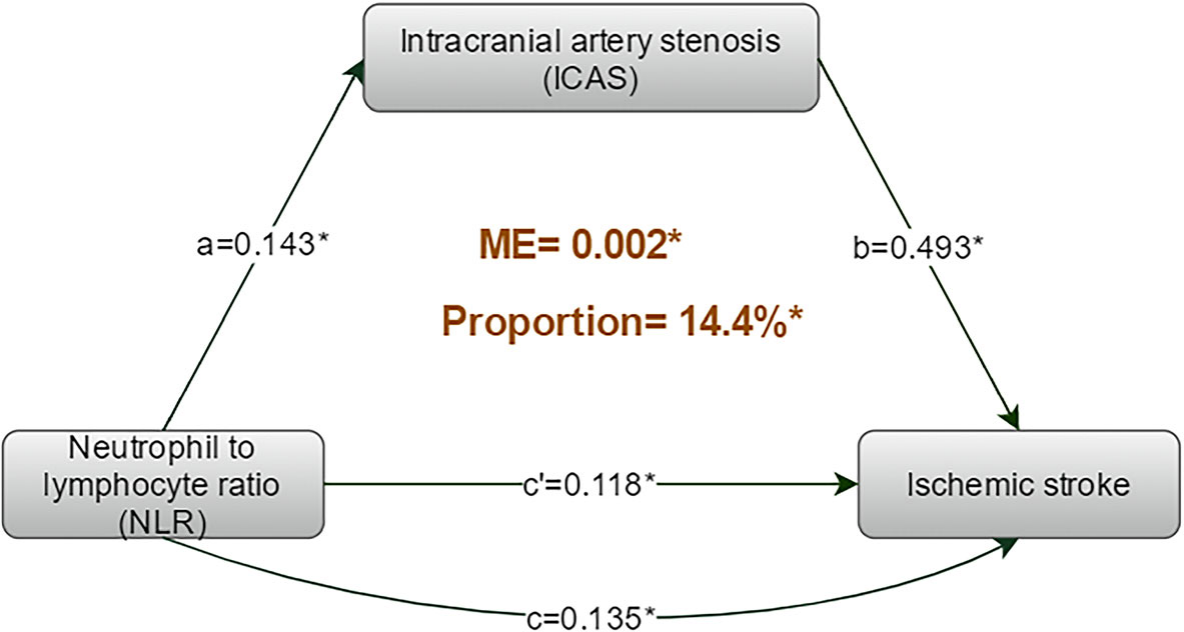
The results of mediation analysis on the risk of ischemic stroke. The mediation analysis showed that ICAS significantly and partially mediate the association between NLR and ischemic stroke, accounting for 14.4% of the total effect (*P* < 0.001). ME: mediated effect; a: the adjusted regression coefficient between NLR and ICAS; b: the adjusted regression coefficient between ICAS and ischemic stroke; c: the adjusted (including ICAS) regression coefficient between NLR and ischemic stroke; c’: the adjusted (barring ICAS) regression coefficient between NLR and ischemic stroke. *p < 0.001

The results of the mediation analysis on the risk of ischemic stroke are depicted in Fig. 2. As TE, NLR significantly associated with ischemic stroke (TE = 0.016, 95% CI 0.010–0.020); within TE, 0.002 (95% CI, 0.001–0.003) was estimated to be an ME; and 0.014 (95% CI, 0.007–0.020) was attributed to the direct effect. From these estimates, the proportion of ME in TE was estimated to be 14.4% (95%CI, 8.0–26.0%): the 14.4% risk of ischemic stroke associated with NLR was mediated by ICAS.

## Discussion

The present study showed that NLR was independently associated with ICAS as well as ischemic stroke in a dose-response pattern. Moreover, we found ICAS significantly and partially mediate the association between NLR and ischemic stroke.

Our results are consistent with recent findings from a cohort study based on a healthy population reporting the independent association of NLR with ICAS [21]. Moreover, several studies reported that high NLR had a strong correlation and precise predictability for restenosis after angioplasty and stenting for asymptomatic carotid stenosis [22]. The mechanisms underlying these observations are not well established, but they seem to be related to the different roles of neutrophils and lymphocytes in the pathophysiologic development of atherosclerosis [23]. As an indicator of acute and chronic inflammation, neutrophil plays a positive role in atherosclerosis [24]. Elevated neutrophil count was reported as a predictor of artery stenosis [25, 26]. A certain number of neutrophils accumulating on the vascular wall can trigger inflammatory reactions through releasing active molecules, such as proinflammatory cytokines and oxyradical, and may lead to endothelial dysfunction and atherogenesis by reducing the utilization of nitrogen oxide [12, 27]. Contrary to neutrophils, lymphocytes have a negative regulatory effect on atherosclerosis. One study indicated that B lymphocytes had protective effects on atherosclerosis caused by hypercholesterolemia [28]. Additionally, lymphocytes are regarded as healing promoters for they secreting anti-inflammatory cytokines interleukin-10 and interleukin-4 [29]. Given their potential roles in plaque formation, it is not surprising that high NLR is significantly associated with ICAS. Besides, both atherosclerosis and ischemic stroke are results of a combination of a variety of risk factors including smoking, alcohol abuse, dyslipidemia, diabetes, and hypertension [30, 31], and several of these risk factors are independently associated with NLR [32–34]. Therefore, NLR may associated with ICAS and ischemic stroke through larger burdens of various vascular risk factors.

Nasr et al’ study has reported significant association of neutrophil count with microembolization in patients with symptomatic carotid artery stenosis [35]. In addition, existing studies points that NLR is significantly correlated with non-calcified plaques [36, 37], which are less stable and more rupture-prone than calcified plaques. Moreover, evidence from human atherosclerosis specimens and murine models of atherosclerosis suggests the presence of neutrophils and neutrophil-derived mediators in atherosclerotic lesions [38, 39]. All these evidences indicate that NLR may participant in plaque rupture, which can well explain the results of mediation analysis in the present study. For one thing, in addition to the mechanisms mediated by active molecules released from neutrophils, novel aspects of neutrophil biology may also contribute to ischemic brain injury. Activated neutrophils have been recently described as components of neutrophil extracellular traps (NETs), which are web-like extracellular scaffolds consisting of DNA, histones, and specific granule proteins, such as neutrophil elastase and myeloperoxidase, in response to various stimuli [40]. Recent evidence indicates that NETs may serve harmful contributors during myocardial and liver ischemia/ reperfusion (I/R) injury and the absence of NETs offers significant cardioprotective, hepatoprotective, and anti-inflammatory effects [41]. These results suggest that NETs may play a role tissue damage. For another, lymphopenia (decreased lymphocyte counts in circulation), resulting from elevated corticosteroids under stress conditions in atherosclerosis patients [42], may cause a weakened healing effects on atherosclerosis.

The NLR reflects the balance between neutrophil and lymphocyte levels, which may be comprehensively represent the immunological conditions; using NLR rather than leukocyte count is the specialty of our study. There were still several limitations. First, we cannot rule out the possibility that there were patients with congenital ICAS and arterial dissection in our subjects. At the same time, 3D MRA may not precise enough to evaluate ICAS. Second, the enrolled participants may all have atheromatous intracranial stenosis vs other causes, which is linked to Asian geographical origin of the study population; on this point, we should explain the present results with caution in other population. Third, although we have considered many traditional risk factors, there are still some confounding factors that are not included in this study. Fourth, we could not conclude the causal relationship between NLR and ICAS as well as ischemic stroke, because the level of NLR was not assessed before the onset. So, more conclusive data from well-designed longitudinal studies are needed to understand the mechanism and long-term effect of NLR on the ICAS and ischemic stroke.

## Conclusions

To summary, the current study demonstrated that NLR was significantly associated with ICAS and ischemic stroke in a dose-response pattern; ICAS partially mediated the association between NLR and ischemic stroke.

## Data Availability

The datasets used and analysed during the current study available from the corresponding author on reasonable request.

## Abbreviations

NLR: Neutrophil to lymphocyte ratio
ICAS: Intracranial artery stenosis
MRI: Magnetic resonance imaging
MRA: Magnetic resonance angiography
CHD: Coronary heart disease
OR: Odds ratio
CI: Confidence interval
TE: Total effect
ME: Mediated effect
SBP: Systolic blood pressure
DBP: Diastolic blood pressure
LDL: Low-density lipoprotein
TC: Levels of total cholesterol
TG: Triglyceride
NETs: Neutrophil extracellular traps
